# Attractive toxic sugar baits for controlling mosquitoes: a qualitative study in Bagamoyo, Tanzania

**DOI:** 10.1186/s12936-018-2171-2

**Published:** 2018-01-10

**Authors:** Marta Ferreira Maia, Frank Chelestino Tenywa, Hannah Nelson, Athumani Kambagha, Abigail Ashura, Ibrahim Bakari, Deogratis Mruah, Aziza Simba, Ally Bedford

**Affiliations:** 1 KEMRI-Wellcome Trust Research Programme, P.O. Box 230, Kilifi, 80108 Kenya; 2 Swiss Tropical and Public Health Institute, Socinstr. 57, 4002 Basel, Switzerland; 3 University of Basel, St. Petersplatz 1, 4002 Basel, Switzerland; 4 Ifakara Health Institute, P.O. Box 74, Bagamoyo, Pwani United Republic of Tanzania; 5 CIVICUS-World Alliance for Citizen Participation, 25 Owl Street, 6th Floor, Johannesburg, South Africa; 6 International Development Consultants, Ground Floor 369 Kilwa House, Toure Drive, Oyster Bay-Kinondoni, P.O. Box 23197, Dar es Salaam, United Republic of Tanzania

**Keywords:** Attractive-toxic sugar bait, Sugar-feeding, Mosquitoes, Qualitative, Focus group discussions, Tanzania, Malaria, Ivermectin, Views and perceptions, Community-based

## Abstract

**Background:**

Malaria elimination is unlikely to be achieved without the implementation of new vector control interventions capable of complementing insecticide-treated nets and indoor residual spraying. Attractive-toxic sugar baits (ATSBs) are considered a new vector control paradigm. They are technologically appropriate as they are simple and affordable to produce. ATSBs kill both female and male mosquitoes attracted to sugar feed on a sugary solution containing a mosquitocidal agent and may be used indoors or outdoors. This study explored the views and perceptions on ATSBs of community members from three Coastal Tanzanian communities.

**Methods:**

Three communities were chosen to represent coastal urban, peri-urban and rural areas. Sensitization meetings were held with a total of sixty community members where ATSBs were presented and explained their mode of action. At the end of the meeting, one ATSB was given to each participant for a period of 2 weeks, after which they were invited to participate in focus group discussions (FGDs) to provide feedback on their experience.

**Results:**

Over 50% of the participants preferred to use the bait indoors although they had been instructed to place it outdoors. Participants who used the ATSBs indoors reported fewer mosquitoes inside their homes, but were disappointed not to find the dead mosquitoes in the baits, although they had been informed that this was unlikely to happen. Most participants disliked the appearance of the bait and some thought it to be reminiscent of witchcraft. Neighbours that did not participate in the FGDs or sensitizations were sceptical of the baits.

**Conclusions:**

This study delivers insight on how communities in Coastal Tanzania are likely to perceive ATSBs and provides important information for future trials investigating the efficacy of ATSBs against malaria. This new vector control tool will require sensitization at community level regarding its mode of action in order to increase the acceptance and confidence in ATSBs for mosquito control given that most people are not familiar with the new paradigm. A few recommendations for product development and delivery are discussed.

## Background

Malaria across endemic regions of sub-Saharan Africa is declining [1, 2], mainly thanks to vector control interventions, such as long-lasting insecticide-treated bed nets (LLINs) and indoor residual spray (IRS) [3]. Despite the success achieved so far, elimination is challenging and the shortfalls of the current interventions need to be addressed with new and complementing vector control tools. Attractive-toxic sugar baits (ATSBs) are considered a new vector control paradigms that kills both female and male mosquitoes [4–10]. The concept exploits the sugar feeding behaviour of mosquitoes, attracting them to sugar feed from a source containing an insecticidal ingredient. The bait targets mosquitoes when they are sugar feeding rather than host seeking or resting, which are traditionally targeted by LLINs, IRS and ATSBs.

The hypothesis is the ATSBs could be an appropriate way to complement existing vector control interventions as they are low-tech, low-maintenance and very affordable. Whereas scientific evidence on the efficacy and effectiveness was proven in controlled conditions, field trials are required to understand how local populations would perceive them. This is especially the case ATSBs as they do not provide direct personal protection but rely on wider effects on the mosquito population to reduce human-vector contact. The current study describes the first social study aiming at describing views and perceptions of community members from Coastal Tanzania on the use of ATSBs.

## Methods

**Fig. 1:**
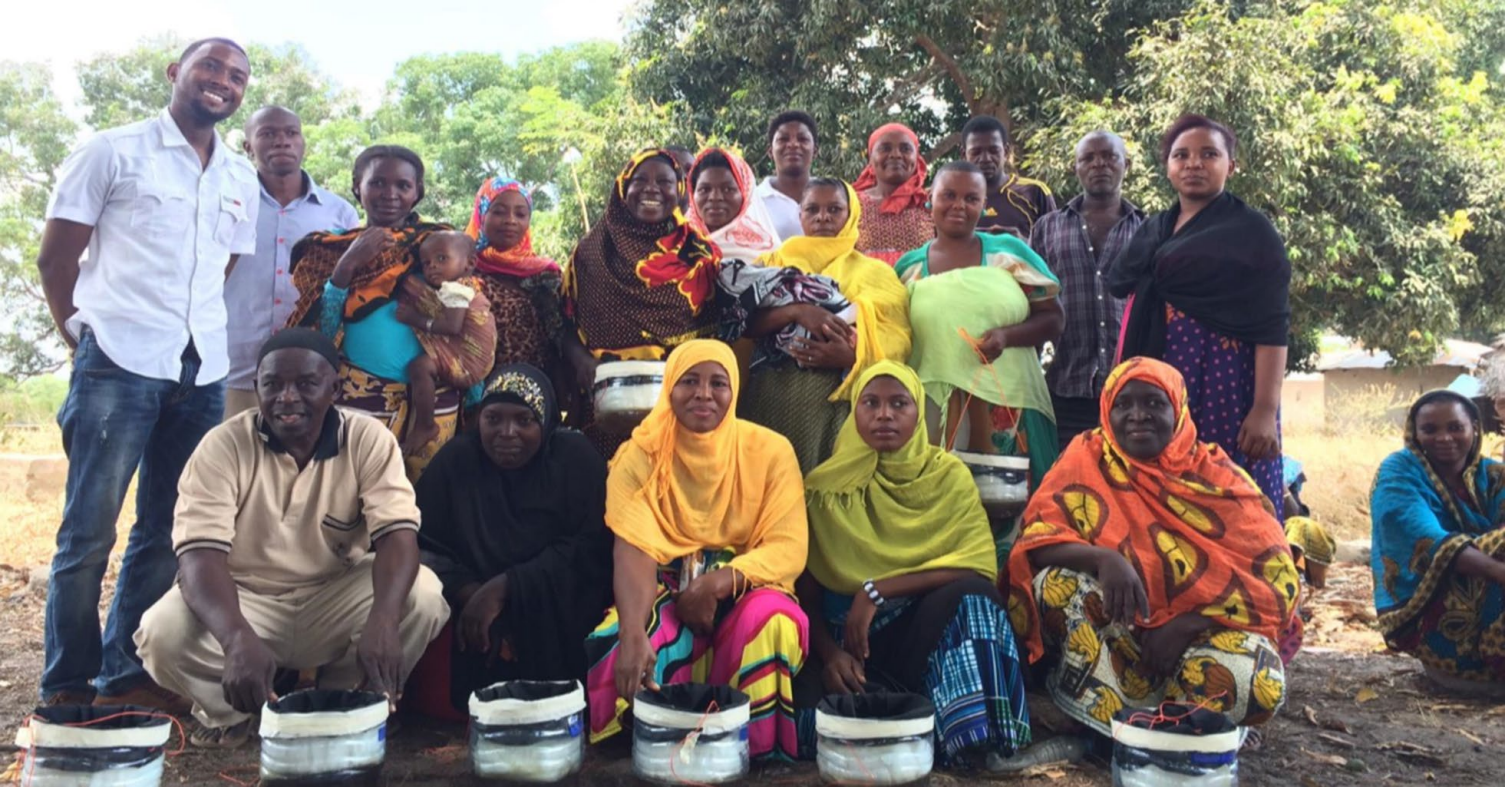
Group of participants who joined sensitization meeting in Kiromo. In front of them are the ATSBs

An attractive-toxic sugar bait was developed and tested inside a biodome at the Ifakara Health Institute [11]. The prototype tested in the semi-field demonstrated an ability to attract at least 50% of the free flying *Anopheles arabiensis* and that these were most likely to choose to feed on a sugar bait placed outdoors amidst vegetation although it would also feed indoors if humans were protected by a bed net [11]. The ATSBs that were distributed contained 0.01% ivermectin in 10% sugar solution, and its structure was made using 100% recycled materials readily available in rural Tanzania (Fig. 1).

Three wards of Bagamoyo district, in the Pwani Coastal Region of Tanzania were selected to participate in the study: Dunda, Kerege, and Kiromo. The villages in each ward represented a range of urban, peri-urban, and rural communities within the area. Dunda covers much of the main town of Bagamoyo (urban) as well as the fishing village of Kaole, Kiromo is situated 10 km south of Bagamoyo town and is mainly composed of small traders and farmers (rural) and Kerege is 25 km south of Bagamoyo with busy roadside commerce and outlying agricultural areas nearing Dar es Salaam (peri-urban). Twenty individuals from each village were invited to participate in sensitization and focus group discussions (FGDs) regarding the ATSBs. Gender was mainstreamed by separating men and women, the methodology also avoided inhibiting women’s opinions.

Sensitization meetings were held in each village before distributing the ATSBs. During the meetings communities were introduced to the concept of attracting mosquitoes to sugar-feed to kill them and were explained how the ATSB worked. All questions, concerns and opinions were answered and recorded. During sensitizations, participants were shown how the bait could be made using basic materials commonly found in household waste. Materials used included: a plastic water bottle (12 L) cut in half, sponge, black cloth, water, sugar, injectable ivermectin solution (IVOMEC^®^ 1%) and string.

The production of an ATSB was demonstrated during the sensitization meeting so that participants could understand how they were made and they were advised to place the baits outside close to vegetation. This has been demonstrated as the best peri-domestic location to place the baits in semi-field experiments and maximize the likelihood of mosquitoes sugar-feeding on the ATSB [11]. The bait had a string tied around it, making it easy to hang and place out of reach from small children or pets. Participants were asked to check that the bait’s sponge was kept moist. Participants were instructed if it dried up to add enough water to soak the sponge but not immerse it. Participants were also informed that the baits did not act immediately, mosquitoes would take 24–28 h to die after ingesting the sugar solution. In addition, it was mentioned that the purpose of this study was not to measure changes in the population of mosquitoes, or longevity of the baits, but rather to understand the practical experience of community members using an ATSB and to receive feedback on how to improve and guide future research. At the end of the sensitization meeting, one ATSB was given to each of the participants and 2 weeks later the community met again for FGDs to ascertain the feedback following the roll-out of the baits. During each session guiding questions were developed to keep discussions on track and to ensure useful feedback was collected. FGDs were documented and common responses were identified and clustered.

## Results

A total of sixty individuals, twenty from each village, showed up to the community sensitization meeting on ATSBs. These individuals were asked to return to participate in FGDs after using the ATSB for 2 weeks. Only 48 participants showed up to the second meeting, most of them were women (Table 1). Frequently asked questions and comments from the sensitisations closely mirrored the type of feedback received in the focus group discussions.

**Table 1:** Number of individuals who participated in sensitization and focus group discussions by gender and village

Session	Sensitization meetings		Focus group discussions	
	Male	Female	Male	Female
Dunda	10	10	7	9
Kiromo	10	10	7	8
Kerege	10	10	7	10
Total	60		48	

### Placement of ATSBs

During sensitization meetings one of the most asked question was related to the placement of the ATSBs. Participants were sceptical about placing the baits outdoors and did not trust they would kill the mosquitoes that bit them if they weren’t placed inside. After 2 weeks, during FGDs, more than 50% of the respondents affirmed they had placed the bait outdoors, as instructed but ended up bringing it inside their homes, where they claimed the bait would be more efficient because that is where mosquitoes usually bit them (Table 2). One person claimed that they placed the bait in the corner of his home because it was full of mosquitoes. Another participant affirmed they had placed the bait outside but moved it inside because they were worried it would dry up too quickly. Ten participants placed the baits directly inside, despite the recommendation to place them outdoors, the participants affirmed they were not interested in killing all the mosquitos but only those that bite indoors, which they believed were the only mosquitoes that spread malaria. Other reasons for keeping the baits inside were because participants were living in rented houses with many other tenants and they did not have control of the house yard or plot other than their own rooms. Also, in more urbanized settings like Dunda and Kerege, houses were close-by and without fences, making it difficult to keep baits outside as they feared passers-by would steal or vandalize them. There were also concerns regarding the safety of the baits to children and animals. One man in Dunda hung his ATSB over six feet inside his house to make sure it was out of his children’s reach.

**Table 2:** Views and perceptions of the participants from Dunda, Kiromo and Kerege in Tanzania to the ATSB

ATSB feature	Respondents (n = 48)		
	< 10% (5)	10–50% (6–22)	> 50% (23–48)
General acceptance of the ATBS			x
Acceptance of the appearance	x		
Acceptance of maintenance needs		x	
Acceptance of design and concept			x
Perceived as effective			x
Preferred placement of the baits			
Indoors			x
Outdoors (recommended)		x	

In Kerege, a few participants also reasoned they had preferred to place the bait inside their homes to avoid neighbours mistaking it for witchcraft:

“*I placed my bait near my shop and had to explain to customers that it was not meant for juju or witchcraft. I think this is related to its appearance and the black cloth”*-Female Participant, Kerege

On the hand, one participant said he had moved the bait indoors but ended taking it back outside because the bait attracted a lot of mosquitoes.

### Appearance

The majority of participants did not like the appearance of the baits (Table 2). A few participants in Kerege expressed that the baits were not very attractive and that their neighbours had associated the black cloth used in the bait with witchcraft. Participants suggested using more colourful materials and printing a pattern or icon that could relate the bait to mosquito control. In the more rural village, participants reported that their neighbours did not believe that the bait meant to kill mosquitoes and there was general disbelief amongst those that had not attended the sensitization meetings. In the urban areas, participants reported mixed experiences with neighbours. Many wanted to know what it was and after learning of its function became excited about it. A few community members wanted to borrow the ATSB and use it in their homes. Many participants reported enjoying teaching others about the baits. One participant complained that the baits produced a bad smell and attracted ants. Another comment was that children became afraid of the bait once they learned it killed mosquitoes.

### Design and maintenance

Most participants were happy with the design of the baits and liked that the fact that they were made with locally available materials that were easy to obtain (Table 2). Some deemed that surface area for mosquito feeding in ATSB was too small and suggested using wider containers, which may attract more mosquitoes. Some suggested construction of a structure that could not be easily moved or stolen, something permanent, for example made of concrete. The most commonly mentioned challenge was the need to check if the baits had dried out (Table 2). Most people said it was an annoyance and mentioned they would easily forgot to maintain the bait because of other responsibilities. Also, a few participants moved the bait indoors because they said if they would place them outside they would have difficulty in maintaining the level of the solution because of the exposure to intense sunshine and/or rain.

### Perceived efficacy

During community sensitization meetings, it was explained how the ATSB works and emphasized that they will not find dead mosquitoes in the baits as the toxicant typically kills the mosquitoes within 24–48 h and not immediately. Despite this, participants were disappointed that they did not see dead mosquitoes. Most participants mentioned that they did not see any difference in mosquitoes when the baits were placed outside, but did notice a reduction when they placed the baits inside (Table 2). One woman expressed a noticeable change in mosquitoes in her room after placing the bait inside:

*“Every night when I sleep I can hear the mosquitoes flying around. Once I moved my bait inside, I did not hear them anymore, even though I still did not find any dead mosquitoes in the bait”*-Female Participant, Kiromo

One participant reported that he would see mosquitoes in the baits in the mornings, but when he touched the bait, all would fly away, making him unsure if the bait was working. However despite this over 50% of the participants perceived the bait as effective (Table 2) and were interested in continuing to use the baits after the project was terminated.

### Sustainability and delivery

One of the topics covered in sensitizations was the sustainability of ATSBs. Participants were asked to think of ways that a program, if implemented, could be effective in their communities. Almost all communities suggested some variation of the same strategy, which would be to train members of the community on how to make the ATSBs and form groups to share costs among members. Most mentioned using the field trial participants themselves as the “ambassadors” or trainers for an initial roll-out. The rationale behind the groups was to offset the costs of making the traps by sharing the costs of materials they needed to buy, i.e. sugar and ivermectin (IVOMEC^®^ 1%). They would also be able to share recycled materials, such as cloth, sponge, rope, or buckets/12 L bottles. Participants emphasized that the first step would be teaching the community about the baits so people would understand how they work, and the importance reducing mosquitoes in their environments. Participants were enthusiastic and mentioned that they were interested in making more baits for themselves and their family members.

## Discussion

Attractive-toxic sugar baits are being promoted as one of the new vector control paradigms. Although there is potential that ATSBs might fill a gap in regards to controlling vectors that LLINs and IRS might fail at, but there is also a fear that the intervention will be badly accepted by the public. Opposed to LLINs and IRS, ATSBs do not directly protect users from mosquito bites and do not trap mosquitoes either. It is difficult for users to perceive a benefit from ATSBs, and thus these may not be accepted and deemed as useless. Current research in the field shows potential of the intervention to reduce mosquitoes by placing ATSBs indoors [12], community members generally also preferred to place the ATSB inside as perceived benefits were low when the bait was placed outdoors. Results from biodome experiments showed that *An. arabiensis* mosquitoes are more likely to feed on baits placed outside amongst vegetation but will also, to some extent, feed indoors provided people are sleeping under a bed-net [11]. It is possible that best results would be obtained if both approaches are followed and this way community members are satisfied to see a measurable effect on their indoor mosquito densities but the full potential of the intervention is not wasted. It would be appropriate to brief communities on mosquito behaviour prior to trials and why the ATSBs are more effective outside than in.

Baits were generally well-accepted by the FGD participants despite other community members, who were not participating in the study, expressing wariness that such a thing would not be able to kill mosquitoes. The baits were linked to witchcraft, which is a common belief and practice in communities of Coastal Tanzania, and particularly Bagamoyo. There was consensus that the bait needed a logo or icon that would associate it with mosquito control. Associations with witchcraft will be dependent on the area where the ATSBs are rolled out as well as the ethnicity of the target population. It is likely that other communities in different geographical areas would not make this association. Participants also disliked having to maintain the bait by keeping it moist. Further product development must evaluate ways of keeping the bait moist with minimal care to ensure compliance is maximized by making the bait as low-maintenance as possible.

Community members also expressed concerns regarding toxicity of the baits and what effect it could have on their children in case they ingested the solution. The baits that were provided in this study contained 0.01% ivermectin, which is a common dewormer used in mass-drug administration against onchocerciasis and filariasis, it is also a very popular drug in the veterinary market. For a small child of 15 kg to ingest a therapeutical dose of ivermectin (200 μg per kg), he or she would have to ingest at least 30 mL of the sugar solution contained in the bait. Since the amount of bait required to make the sponge mattress in the prototype wet was 1 L, it would require 10 mL of 1% ivermectin. This dose is greater than the minimum dose recommended for child weighing 15 kg. To count this the design used in the ATSB trapped the solution in a sponge making it difficult to retrieve such an amount without completely dismantling the bait and squeezing the sponge. However, to make the intervention safe for community use grilling or hanging them should be recommended when it comes to the field application. Nonetheless, individuals pro-actively addressed their own concerns by hanging the baits out of children’s reach. Future product development must choose a toxicant with very low or negligible mammalian toxicity. ATSBs in other studies have been commonly tested using 1% boric acid solution [7–9, 12, 13]. Investigators using boric acid in their ATSB formulations should reconsider the use of this compound as it is toxic to mammalian reproduction. If a 2-year-old child consumes sugar solution from a bait containing 1% boric acid, it can be exposed to a dose potentially causing future reproductive complications by only ingesting 0.1 mL of solution [14].

Although it was not the projects objective to measure efficacy of the bait at controlling mosquitoes it was clear that many participants felt they should report they heard fewer mosquitoes inside their homes after moving the baits inside. One participant complained that the bait attracted mosquitoes and these would be seen resting on the walls of the baits but would not be dead. This is consistent with how an ATSB is designed, seeing mosquitoes inside the bait should be regarded as a confirmation that the bait is working. However, a few participants stated they perceived a reduction in mosquitoes within their homes but were disappointed that they didn’t find dead mosquitoes.

The use of recycled materials to produce the ATSBs sparked mixed opinions, although few people regarded the look as shabby and unofficial, most of the participants were pleased with being able to produce the bait themselves rather than buying them or rely on officials to distribute them. A logotype should be incorporated in the design that easily associates the bait with mosquito control. Participants agreed that the way towards reaching sustainability must actively involve the community to create awareness about this new vector control paradigm. In addition, a Training of Trainers (TOT) model was suggested in which programme resources could be used to train and inform individuals about ATSBs, supported by a larger community group who would share the cost of the items that need to be bought or collected. A TOT model would generate a pool of human resources and contribute to capacity building in communities where large proportions may be unemployed, this would lead to members gaining a sense of accomplishment if community recognizes a long-term gain in the intervention by noticing fewer mosquitoes in their homes. However it is questionable if this approach is realistic and if an affordable commercial product supported by a community engagement program wouldn’t be a better option.

## Conclusions

This study provides extensive information on how communities of Coastal Tanzania perceive ATSBs. The information summarized here will be very useful for researchers designing future trials aimed at assessing the clinical and epidemiological benefits of using ATSBs in regards to malaria transmission. The ATSBs appearance was important to community members. More than 50% of the respondents disliked the shabby nature of a recycled-based bait and preferred if the baits would have a more official and colourful appearance that could easily associate it with mosquito control. Participants preferred placing the baits indoors because they believed most mosquitoes were indoors. Compliance with keeping a functional ATSB might be low if it needs frequent maintenance. Community members might not accept the new tool because they are unaware of how it works and are sceptical of its efficacy. It was noted that, after sensitization, participants understood the concept of ATSBs and were likely to accept the new way of killing mosquitoes. The introduction of ATSBs as a novel vector control paradigm in Tanzanian communities would require extensive community sensitization to avoid scepticism around the new concept and to ensure communities understand how the baits work and accept them in their daily lives.
